# Potential Distribution of *Aedes* (*Ochlerotatus*) *scapularis* (Diptera: Culicidae): A Vector Mosquito New to the Florida Peninsula

**DOI:** 10.3390/insects12030213

**Published:** 2021-03-03

**Authors:** Lindsay P. Campbell, Nathan D. Burkett-Cadena, Evaristo Miqueli, Isik Unlu, Kristin E. Sloyer, Johana Medina, Chalmers Vasquez, William Petrie, Lawrence E. Reeves

**Affiliations:** 1 Florida Medical Entomology Laboratory, Department of Entomology & Nematology, IFAS, University of Florida, 200 9th St SE, Vero Beach, FL 32962, USA; nburkettcadena@ufl.edu (N.D.B.-C.); ksloyer@ufl.edu (K.E.S.); lereeves@ufl.edu (L.E.R.); 2 Broward Mosquito Control Section, 1201 W Airport Rd., Pembroke Pines, FL 33024, USA; EMIQUELI@broward.org; 3 Miami-Dade Mosquito Control Division, 8901 NW 58 St., Miami, FL 33178, USA; isik.unlu@miamidade.gov (I.U.); johana.medina@miamidade.gov (J.M.); chalmers.vasquez@miamidade.gov (C.V.); william.petrie@miamidade.gov (W.P.)

**Keywords:** invasive species, *Aedes*, ecological niche models, species distribution models, vector surveillance

## Abstract

**Simple Summary:**

*Aedes scapularis* is an important mosquito species capable of transmitting viruses and parasites to humans and animals. *Aedes scapularis* was previously known to occur throughout large portions of the Americas, from the lower Rio Grande Valley of southern Texas to Argentina and on several Caribbean Islands. Recently, this mosquito became established in southern Florida, marking the first time *Ae. scapularis* was found on the Florida Peninsula. Now that *Ae. scapularis* has reached the Florida Peninsula, it is expected to continue to expand its geographic distribution to fill contiguous areas with suitable environments. Here, we use a modeling approach that correlates environmental variables with known geographic collection locations of *Ae. scapularis* to predict the potential distribution of this species. The output of this model provides new information for mosquito control and public health agencies to help monitor the spread of this exotic vector mosquito and suggests a need for surveillance for the expansion of this mosquito in many of Florida’s coastal counties.

**Abstract:**

*Aedes scapularis* is a neotropical mosquito known to transmit pathogens of medical and veterinary importance. Its recent establishment in southeastern Florida has potential public health implications. We used an ecological niche modeling approach to predict the abiotic environmental suitability for *Ae. scapularis* across much of the Americas and Caribbean Islands. Georeferenced occurrence data obtained from the Global Biodiversity Inventory Facility and recent collection records of *Ae. scapularis* from southern Florida served as input for model calibration. Environmental layers included bioclimatic variables provided in 2000 to 2010 average Modern Era Retrospective-analysis for Research and Applications climatic (MERRAclim) data. Models were run in the software program Maxent. Isothermality values often found in costal environments, had the greatest contribution to model performance. Model projections suggested that there are areas predicted to be suitable for *Ae. Scapularis* across portions of the Amazon Basin, the Yucatán Peninsula, the Florida Peninsula, and multiple Caribbean Islands. Additionally, model predictions suggested connectivity of highly suitable or relatively suitable environments spanning the United States Gulf Coast, which may facilitate the geographic expansion of this species. At least sixteen Florida counties were predicted to be highly suitable for *Ae. scapularis*, suggesting that vigilance is needed by vector control and public health agencies to recognize the further spread of this vector.

## 1. Introduction

Changes in the geographic distributions of medically important vector mosquitoes can result in broad-scale negative impacts on human and veterinary health [1,2]. Most notable is the human-mediated dispersal and subsequent establishment of *Aedes aegypti* L. and *Aedes albopictus* Skuse in multiple regions of the world, facilitating the spread of dengue, yellow fever, chikungunya, and Zika viruses into new geographic areas [1,3,4,5,6,7]. Although often overlooked, finer scale changes in the geographic distributions of mosquito vectors can also alter transmission risk, warranting close monitoring of local mosquito faunas for introductions of non-native mosquito species, and for the potential of introduced species to contribute to the transmission of mosquito-borne pathogens [8]. Further, anticipated changes in global climate may lead to shifts in the geographic, elevational, or seasonal occurrence of mosquito species, prompting concerns of increased uncertainty in potential transmission risks of mosquito-vectored pathogens [9].

Several factors facilitate or constrain geographic distributions of mosquito vector species [10,11], including the presence of geographic barriers to dispersal, temperature and humidity constraints, and the availability of suitable larval habitats. Importantly, biotic variables, including competition, predation, and resource availability in a specific area, can influence species distributions at relatively fine scales, while abiotic climatic values can serve as broader scale constraints from which to characterize the potential distribution of a species [12,13,14,15]. Mosquitoes, as insects, are ectotherms, making them sensitive to temperature, while their small body size makes them vulnerable to low humidity [16]. As such, abiotic climate variables have been used extensively to predict potential distributions and changes in distributions of medically important vector arthropod species and pathogens. For example, multiple global scale models have predicted the potential distribution and changes in the potential distribution of globally invasive *Aedes aegypti* with climate change [17,18,19,20] and in South America, abiotic variables were included in a model predicting the potential distribution of *Lutzomyia longipalpis* [21]. In North America, abiotic variables were used to predict the potential distribution of *Aedes japonicus*, a medically important and recent invasive species to this continent [22].

The geographic location of mainland Florida, USA, spanning multiple ecoregions and climatic gradients [23], along with its diversity of habitats, proximity to several Caribbean islands and high levels of trade and tourism, makes this area a prime candidate for the invasion and establishment of mosquito vector species from the Neotropics and elsewhere [24]. Importantly, of Florida’s 16 non-native and suspected non-native mosquito species, 13 (81.3%) were first detected in the state since 1985, and 10 (62.5%) were first detected in the last 20 years.

The recent expansion in the geographic distribution of *Aedes scapularis* Rondoni onto peninsular Florida presents a new challenge to vector management and mosquito control programs [25]. *Aedes scapularis* is an important vector mosquito in the American Tropics [26]. A diverse assemblage of arboviruses and parasites have been detected in wild females, including flaviviruses (yellow fever, Rocio, and Ilhéus viruses [27,28,29,30]), an alphavirus (Venezuelan equine encephalitis virus [31,32,33,34]), an orbivirus (Yunnan orbivirus [35]) and filarial nematodes (*Dirofilaria immitis* [36] and *Wuchereria bancrofti* [37]). *Aedes scapularis* is considered a generalist in its use of habitats, occurring in both sylvatic and human-dominated areas at low and middle elevations. The larvae develop in temporary pools filled by rainfall or overflowing waterways [26]. Adult females are considered opportunistic in their host use, though they feed frequently from endothermic hosts, especially mammals [25,38,39,40,41,42,43,44,45]. Humans are frequent hosts for female *Ae. scapularis*, and in some human dominated areas, the species shows synanthropic adaptations such as readily entering buildings, and host-seeking and blood-feeding indoors [46,47]. The wide host breadth and frequent use of human hosts coupled with synanthropic adaptions suggests that *Ae. scapularis* may be well positioned, ecologically, to serve as a bridge vector for human and animal pathogens [25], highlighting further the need to utilize robust tools to monitor the expansion of this species in the Florida Peninsula.

Prior to its recent range expansion into the Florida Peninsula, the known geographic distribution of *Ae. scapularis* comprised large portions of South and Central America, southern and eastern Mexico, and several Caribbean islands, outlined in a map produced by Arnell [26] plotting the approximate collection locations of examined *Ae. scapularis* specimens. As the recent observations of *Ae. scapularis* indicate a northward expansion in the geographic distribution of this species onto the contiguous US mainland, a need exists to update and to characterize its potential distribution to help inform future veterinary and public health surveillance and control efforts.

In Florida, *Ae. scapularis* is currently established in Miami-Dade and Broward Counties [25]. Mark-recapture studies indicate that adult female *Ae. scapularis* can disperse relatively large distances, up to 4.1 km [48], making it likely that the geographic distribution of *Ae. scapularis* on the Florida Peninsula will expand to fill adjacent suitable environments over time. Ecological niche modeling, or species distribution modeling, is a correlative modeling approach that utilizes environmental data collected at georeferenced locations where a species has been observed to predict where similar combinations of environments occur across a broader geographic area, assuming that the species is in equilibrium with its environment [49]. Predicting the potential distribution of vector species provides a useful tool to help target monitoring and surveillance efforts, contributing to more efficient vector control and public health management strategies [50]. Here, we use ecological niche modeling to predict the potential distribution of *Ae. scapularis* incorporating recently published georeferenced records from the southern Florida Peninsula.

## 2. Materials and Methods

Georeferenced *Ae. scapularis* occurrence data were downloaded from the Global Biodiversity Inventory Facility (GBIF.org (21 October 2020) GBIF Occurrence Download https://doi.org/10.15468/dl.j5y6ua, (accessed on 21 October 2020) and combined with *Ae. scapularis* records collected in southern Florida [25]. Data without geographic coordinates, and occurrence records for which the precision of the decimal degree was less than three decimal places were removed from the data set to allow moderate flexibility in the precision of the coordinates, given the intended 2.5′ (i.e., ~5 km) spatial resolution of our model. Georeferenced occurrence data were then mapped and thinned spatially at a 0.25 decimal degree distance using functions available in the “ntbox” package in R [51] to help prevent overrepresentation of environmental combinations owing to sampling bias and to help reduce potential impacts from spatial autocorrelation on model calibration [52]. Additionally, we extracted a set of environmental values at each location and calculated the Mahalanobis distance, which is a multivariate distance measure, to identify whether environmental thinning would be needed to reduce redundancy in combinations of environments in our data set, which can lead to biased model calibration [53].

### 2.1. Calibration Area

**Figure 1:**
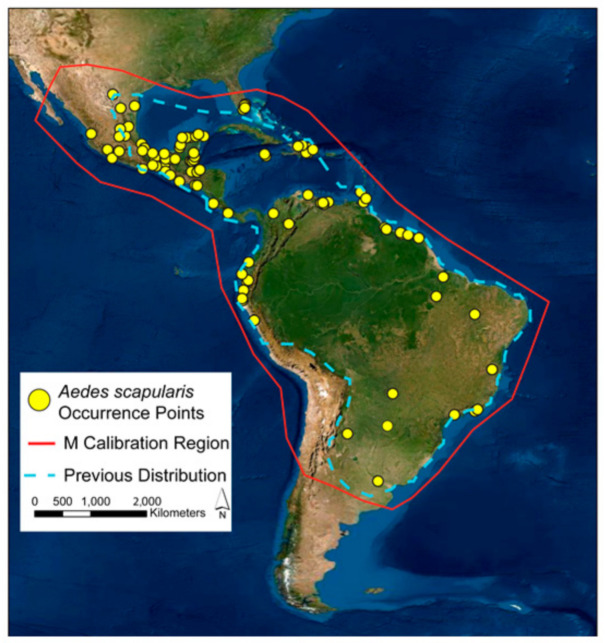
Distribution of *Aedes scapularis* georeferenced occurrence points used in model calibration and evaluation (yellow points); red polygon represents the M calibration region. Blue, hashed-line polygon indicates previously recognized distribution of *Ae. scapularis*, redrawn from Arnell (1976). Base map provided by Environmental Systems Research Institute.

A major component in ecological niche modeling is delineation of the user-defined model calibration region [54]. Multiple approaches are used to delineate the user-defined calibration region, including calculations of convex hulls surrounding occurrence records, usually with the inclusion of user-defined buffer distance, and alpha-shapes [55]. Here, we followed the conceptual framework first outlined in [54] and explained in detail in [49] for which the calibration region is described as the area accessible to a species through dispersal over relevant time periods, termed “**M**”. This approach provides the opportunity for model discrimination to include combinations of environments in regions where a species has had access, but does not utilize, providing more useful information in model interpretation. Following the guidelines of this framework, we included in our **M**-calibration region for *Aedes scapularis* the majority of South America, all of Central America, multiple Caribbean Islands, Mexico, and a portion of the southern United States in North America (Figure 1). We limited the extent of the **M**-calibration region in southern South America based on a general transition from warm temperature fully humid environments to arid steppe cold environments following the Köppen−Geiger climate classification system [56], and we limited the extent in western North America excluding the majority of the Sonoran Desert and at the transition to higher elevations; we were conservative in the delineation of the **M**-calibration extent in Florida because *Ae. scapularis* is new to the Peninsula and has had a limited time for dispersal.

### 2.2. Environmental Data

Combinations of bioclimatic variables derived from average temperature and specific humidity variables were acquired at a 2.5′ spatial resolution (~ 5 km) for the years 2000 to 2010 from the Modern Era Retrospective-analysis for Research and Applications climatic data set (MERRAclim) [57]. Multiple climate data sets are available for model calibration and projection for contemporary time periods. The MERRAclim data used here consists of bioclimatic variables derived from satellite-based temperature and specific humidity data collections at an hourly time interval from 1981 to 2010; for a comparison between bioclimatic variables derived from the commonly used WorldClim Bioclimatic data set and MERRAclim see [57]. The MERRAclim bioclimatic variables served as environmental variables in model calibration. Bioclimatic layers were masked to the **M**-calibration region using the ’raster’ package in R v3.6 [58].

Correlation between environmental variables is common when using bioclimatic layers, which can result in redundancy and highly complex models that produce interpretation challenges [59]. We calculated a Pearson’s correlation matrix using environmental values obtained within the **M**-calibration region to identify pairwise correlations between each environmental variable and generated five candidate sets that included variables that were not highly correlated (Table 1 and Table S1, Figure S1).

### 2.3. Model Calibration

Multiple modeling approaches exist for SDM/ENM applications. Here, we implemented a maximum entropy algorithm in the Maxent 3.41 software package [60], executed within the “kuenm” package in R [61]. This approach allowed us to employ a multi-step model evaluation process, including model selection based on an information criterion approach [62], while identifying an optimal regularization multiplier value comprehensive to our data set [61]. Additionally, the most recent version of the Maxent software program implemented here is comparable to an inhomogeneous Poisson point process model, which treats occurrence data as a continuous process, rather than a quadrat area discretized within the pixel size extent [63]. Briefly, the Maxent software algorithm discriminates the range of environments at georeferenced occurrence locations (i.e., presence locations) with the range of environments found at a set of “background” locations, distributed randomly across the calibration area [64,65]. A regularization multiplier helps to control for overfitting, which can reduce predictive performance, contributes to the variable selection process, and reduces redundancy if correlated variables are present [66]. Models were run using a random subset of 70% of the occurrence data with the remaining 30% of the data withheld for model evaluation. Candidate models were generated for each of the five environmental data sets, using a combination of feature classes (i.e., linear [l], quadratic [q], product [p], linear+quadratic [lq], linear+product [lp], quadratic+product [qp], linear+quadratic+product [lqp]) and regularization multipliers ranging between 0.1 and 10. Initial model runs included an internal 50% random subset for training and testing with 10 bootstrap replicates at 500 iterations each, and 10,000 background points.

### 2.4. Model Evaluation

Model evaluation followed a three-step process outlined in Cobos et al. [61]. Calibration results were filtered first to identify models with statistically significant partial area under the curve of the receiver operating characteristic values (pROC) [67], and only models with omission rates < 5% were retained. Filtered calibration results were ranked from lowest to highest using Akaike’s information criterion scores corrected for small sample sizes (AICc) to identify a final candidate set of models [62]. Model projections were then generated from the best performing model identified in the model evaluation process using the joint occurrence data, and the median of 100 bootstrap replicates served as the final model projection using Maxent’s “cloglog” output [63]. Additionally, we investigated the potential for model projections to extrapolate to combinations of novel environments not represented within the **M**-calibration region using the Multivariate Environmental Similarity Surface (MESS) function available in the Maxent software package [68]. Visual inspection of model predictions from the best performing model were compared to the relative locations described in Arnell [26] to identify outstanding distribution questions, gaps in model predictions, and to target future priority sampling areas.

## 3. Results

A total of 781 georeferenced occurrence points were acquired for ecological niche models. After removing duplicates, 166 occurrence points remained, and after spatially thinning the data, 97 occurrence points remained (Figure 1 and Table S2). A comparison of the raw occurrence data and thinned occurrence data are available in Figure S2. A plot of Mahalanobis distances across the data set did not reveal evidence of strong multivariate environmental redundancy, and thus, we did not perform additional environmental thinning of the data (Figure S3). Plots of the Mahalanobis distances did reveal three occurrence locations with combinations of environmental values with greater distances from the majority of the occurrence records. These records included the most southern occurrence record in South America, the most southern occurrence record along the Pacific Coast of South America, and the most northwestern occurrence record along the Pacific Coast of Mexico (Figure S3). Additional inspection of these records revealed that they were submitted from the U.S. Smithsonian National Museum of Natural History and the Instituto Nacional de Diagnóstico y Referencia Epidemiológicos which we regard as reputable (Figure S3).

**Figure 2:**
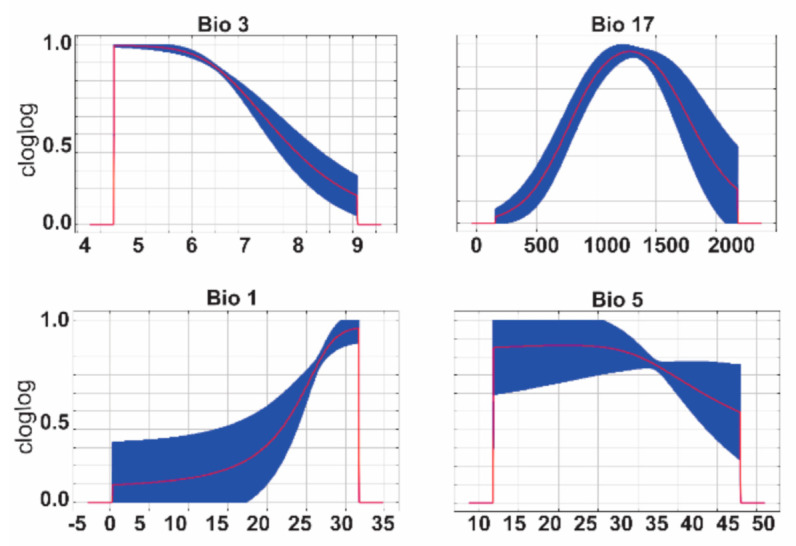
Response curves of bioclimatic variables in the final model. Bio3, Bio1, and Bio5 values are in units of degrees Celsius; Bio17 specific humidity values are units of 100,000 * kg of water/kg of air.

Of these 97 occurrence points, 61 were used for model training and 36 were used for model evaluation. A total of 595 candidate models were generated across the five environmental data sets (Table S3). Model evaluation indicated that two models met the criteria of statistical significance (Table S4), with omission rates < 5%, and the model with the lowest AICc score was chosen as the final model. The best performing model included four bioclimatic data layers, included quadratic and product features, and a regularization multiplier of 0.3 (Figure 2), and an AUC value of 0.80 (Figure S4). Environmental variables in the final model and percent contributions to model performance are outlined in Table 2.

Marginal response curves for Bio3 indicated that environmental suitability decreased as average isothermality increased. For Bio17, results indicated that predicted environmental suitability was optimal at a value of approximately 1000 for average specific humidity of the driest quarter, and results suggested an increase in suitability with increases in Bio1 (average annual temperatures). Response curves for Bio5 indicated a slight decrease in predicted suitability when mean maximum temperature of the warmest month reached approximately 35 °C.

**Figure 3:**
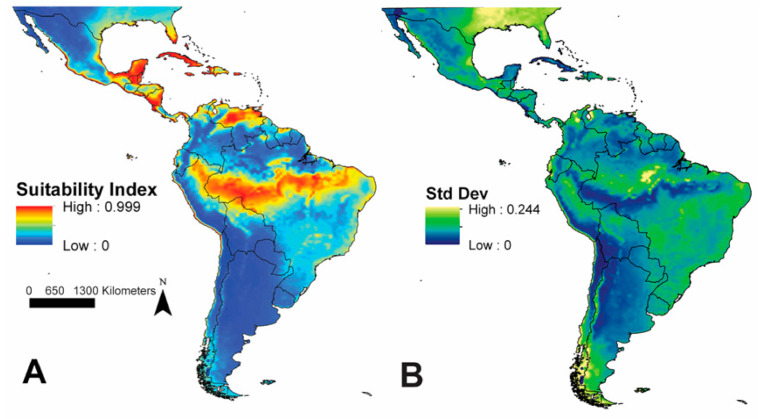
(**A**) Model prediction including the model calibration region and the projection region; red areas indicate high predicted suitability and blue areas indicate low predicted suitability; (**B**) standard deviation of predicted suitability values across 100 bootstrap replicates; light blue areas indicate higher standard deviation values and dark blue areas indicate low standard deviation values.

**Figure 4:**
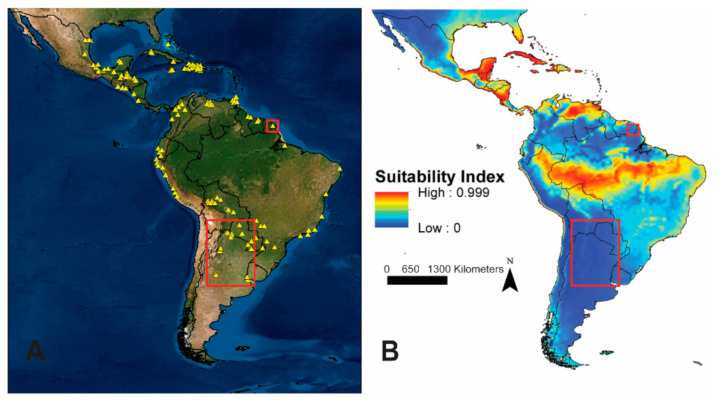
(**A**) *Aedes scapularis* collection records (yellow triangles) reported in and adapted from Arnell 1976. (**B**) *Aedes scapularis* projection model output. Red squares indicate areas of discrepancy where *Ae. scapularis* collections were reported and examined by Arnell (1976) and where model outputs did not predict suitable environments for *Ae. scapularis*.

The model projection predicted several regions to be highly suitable for *Ae. scapularis* across the study region (Figure 3A or Figure 4B). Regions where predicted environmental suitability for *Ae. scapularis* were highest included the Pacific coastline of the Americas, from northern Chile to southern Sonora, Mexico, a broad longitudinal belt across much of South America, along the southern side of the Amazon River, from coastal Brazil west to northern Peru, the llanos of Venezuela around the Orinoco River, the Yucatán Peninsula, southern and Central Nicaragua and the Nicoya Peninsula of Costa Rica, most Caribbean Islands with the exception of the interior of Hispaniola, the Florida Peninsula, and along the Gulf Coast of the United States between Texas and the Florida Panhandle.

Plots of the standard deviation of the 100 replicates indicated relatively low variation across the model outputs for the majority of the study area. Areas with higher standard deviation values included the southwestern tip of Chile in South America, a portion of the Amazon Rainforest in Pará and Amazonas States in Brazil, in Colombia, where elevation values increase at Pico Cristóbal Colón, and then more generally, in the southeastern United States, beginning in central Florida and moving north- and westward (Figure 3B). Inspection for the potential of model extrapolation to novel environments revealed only two small areas within the projection region that had combinations of environments not present within the calibration region. These two areas included the extreme southwestern tip of South America and the extreme northwestern portion of the projection region, just north of the Baja Peninsula (Figure S5).

Other than the southern Florida Peninsula, and areas predicted highly suitable along the coast of the Gulf of Mexico from Florida to Texas and Mexico’s Pacific coast, areas predicted highly suitable were within the previously reported distribution of *Ae. scapularis* (see Figure 1 and Figure 4A) [26]. Comparison of *Ae. scapularis* collection records described in Arnell 1976 (Figure 4A) with model predictions (Figure 4B) indicated a discrepancy in southern South America, where georeferenced occurrence data were not available for our model calibration. Model outputs suggested unsuitable environments for *Ae. scapularis* in this region, but observations were described in Arnell [26]. Additionally, model outputs predicted unsuitable environments in French Guiana, even though Arnell [26] described *Ae. scapularis* in this area and a georeferenced occurrence point was available for model calibration.

**Figure 5:**
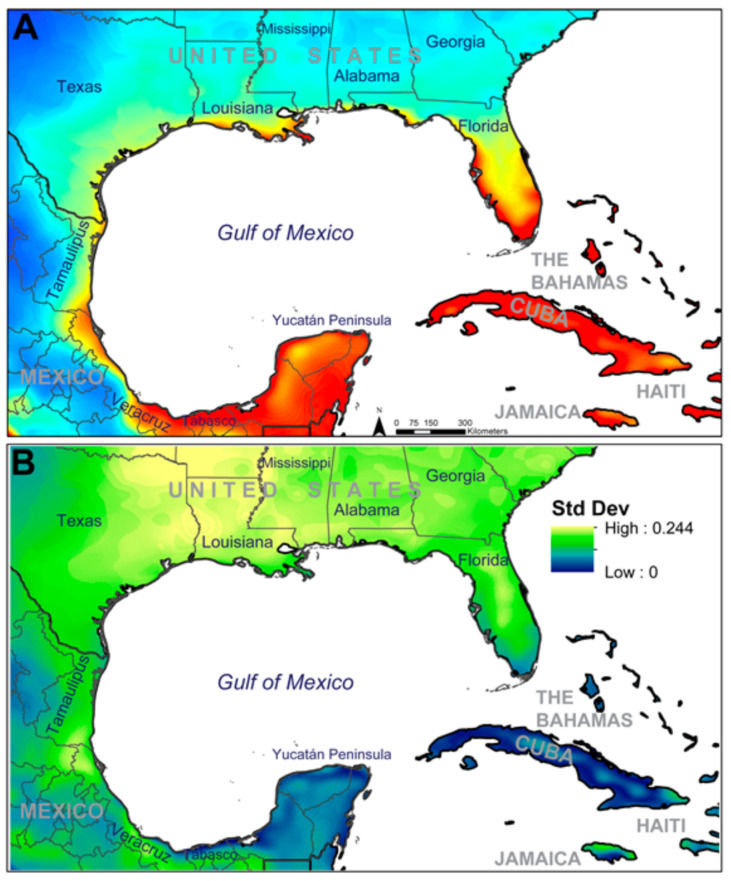
Model projection to Southern United States, Caribbean Islands, and Mexico; (**A**) predicted suitability, (**B**) standard deviation.

Observations of predicted values in North America, beginning in the Yucatán Peninsula and moving northward around the Gulf Coast into the states of Texas, Louisiana, Mississippi, Alabama, and Florida indicated relative connectivity of suitable environments across this region, with areas predicted highly suitable in the Yucatán Peninsula, through Tabasco and Veracruz to Tamaulipas (Figure 5A). Additional areas predicted highly suitable were located along the Gulf Coast of the United States at the southern tip of Texas, across the southern coastal edge of Louisiana including the City of New Orleans, in Gulf County in the Panhandle of Florida, and then along the Gulf Coast of the Florida Peninsula. Areas predicted to be moderately suitable for *Ae. scapularis* are located throughout portions of eastern Texas, Louisiana, inland in Mississippi, southern Georgia, South Carolina, and nearly the entirety of Florida; although, standard deviation values across these areas suggest, in general, relatively high variability in model outputs in these areas (Figure 5B).

**Figure 6:**
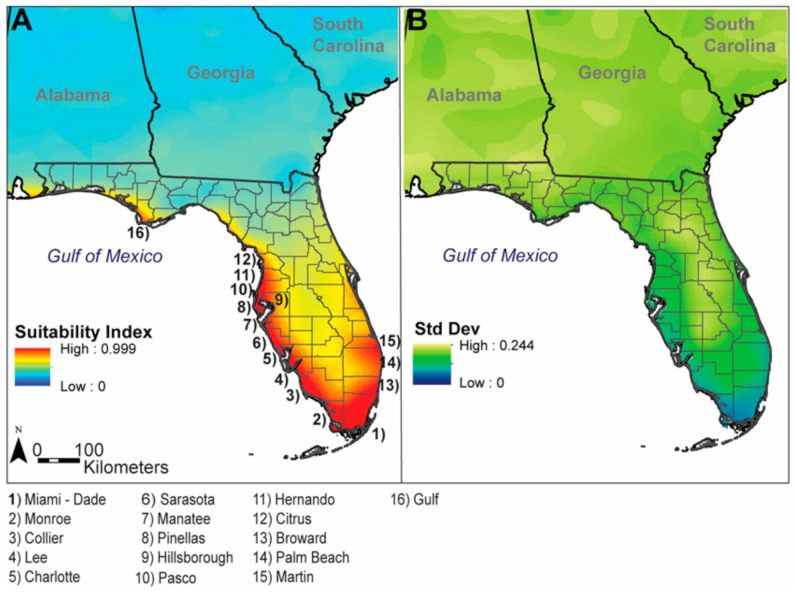
(**A**) Model projection of predicted suitability for *Ae. scapularis* in Florida and surrounding states; (**B**) Standard deviation values in Florida and surrounding states.

Model outputs predicted that much of the Florida Peninsula and Florida Panhandle were relatively suitable for *Ae. scapularis*, with suitability decreasing northward onto the Atlantic Coastal Plain of the United States, with a narrow band of high suitability along much of the United States’ Gulf of Mexico coastline (Figure 6A). Specifically, several Florida counties contained areas predicted highly suitable for *Ae. scapularis*, including Miami-Dade, Broward, Palm Beach, and Martin Counties along the Atlantic Coast, with predicted values decreasing, moving northward into St. Lucie, Indian River, and Brevard Counties. Counties containing areas predicted highly suitable on the Gulf Coast of Florida included Monroe, Collier, Lee, Charlotte, Sarasota, Manatee, Pinellas, and portions of Hillsborough, Pasco, Hernando, and Citrus Counties, with predicted values decreasing continuing northward along the coast of the Big Bend region and onto the Florida Panhandle, before increasing again in Gulf County. Standard deviation values within Florida indicated higher variability beginning in central Florida and moving northward, with greater values in model predictions in the southeastern United States, beginning in central Florida and moving northward and across portions of the Florida Panhandle (Figure 6B). 

## 4. Discussion

The objective of this study was to predict the potential geographic distribution of *Ae. scapularis*, an important vector of multiple arboviral and parasitic diseases. The recent identification of established *Ae. scapularis* populations on the Florida Peninsula indicated a recent expansion in the known geographic range of this species and highlights the need to monitor further geographic expansion by characterizing environments that may be suitable for this species. To our knowledge, this effort is the first distribution model to incorporate georeferenced occurrence data with abiotic environmental variables to predict the potential distribution of this important invasive species.

Here, we used an ecological niche modeling approach to predict the potential geographic distribution of *Ae. scapularis* across much of the Americas and the Caribbean Islands, with emphasis on environments that may be suitable for this species along coastal areas of North America, and more specifically, in the State of Florida in the United States. The best performing model included four environmental variables summarizing temperature and specific humidity values. Model results indicated that locations that did not have extreme ratio values between average diurnal temperature values and overall temperature values, that generally had warmer average annual temperatures, but not extreme maximum temperatures, and relatively average specific humidity values were predicted suitable for *Ae. scapularis*.

Model outputs predicted suitable environments for *Ae. scapularis* matching closely to historical collection localities outlined by Arnell [26] and adapted in Figure 4A, as did the majority of the occurrence records used here for model calibration, with the exception of a few regions for which precise georeferenced occurrence points were not available, and in Florida, where the recent geographic expansion occurred. Arnell’s records and the georeferenced occurrence points used here generally tracked along or near coastal regions with fewer observations present in inland regions, and this phenomenon was evident in the model results, with average isothermality (mean diurnal range/temperature annual range) contributing the greatest to model performance. Model outputs predicted highly suitable environments along several coastal areas where maritime climates with ocean waters that mediate large temperature fluctuations between daylight and nighttime hours are present [69] and in additional regions exhibiting similar isothermality values. These results may highlight the importance of average isothermality values to the survival and reproduction of *Ae. scapularis*, but the possibility also exists that these results may be an indicator of greater sampling effort and accessible sampling opportunities across coastal regions, where higher densities of people were present and the terrain does not present an obstacle to sampling.

While model results predict coastal regions to be highly suitable for *Ae. scapularis*, an important and outstanding question remains regarding the extent to which *Ae. scapularis* utilizes inland environments, given the less frequent but described inland observations. Of particular interest is the extent to which *Ae. scapularis* may inhabit heavily forested environments such as the Amazon Basin. There, our model outputs suggested highly suitable environments south of the Amazon River, where mid-range average isothermality values were present, while predicting low suitability north of the Amazon River, where average isothermality values rise. Model outputs did not predict suitable environments in French Guiana at the transition into the Amazon Forest, despite the inclusion of an occurrence point in this region in model calibration. This result demonstrates the need for additional sampling in areas transitioning into these forested environments. Additionally, many of Arnell’s observations in Paraguay and northern Argentina that were not predicted suitable in our model fall within the “dry chaco” ecoregion, a hot and semiarid lowland natural region of the Río de la Plata Basin that has a long (6-month) dry season. Predicted suitability was also low further south in the Province of Río Negro, Argentina [70], where the climate is likely at the extreme limits of minimum temperature tolerance for this species. A lack of precise or sufficient number of georeferenced occurrence records representing the combination of environments in French Guiana and at these inland locations in model calibration likely contributed to this outcome, demonstrating one limitation of this modeling framework. These results highlight the benefit of continued and updated georeferenced data to inform model outputs. Considering that *Ae. scapularis* occurs across a broad temperate area in the southern portion of its range, cool winters on the central and northern Florida Peninsula may not inhibit northward expansion of this species’ geographic distribution. As *Ae. scapularis* continues to expand its geographic range, and additional georeferenced occurrence points become available, we anticipate the potential for an increase in inland occurrence data and that these additional data points may alter the contribution of environmental variables to model performance and predictions.

Model predictions suggested highly suitable or relatively suitable environments for *Ae. scapularis* along much of the Gulf Coast of the United States. Importantly, no major gaps in highly suitable or relatively suitable values occurred across this area, suggesting that the environments in this region could serve as an environmental corridor for continued range expansion throughout the region. Although we emphasize the introduction of *Ae. scapularis* in southern Florida, connectivity of suitable environments along the Gulf Coast could also facilitate movement of this species between northern Mexico and the Florida Panhandle. Since 1916, *Ae. scapularis* has been known from southernmost Texas (Cameron and Hidalgo Counties) [26], but to our knowledge, no additional published records from elsewhere in Texas exist to indicate its distribution has expanded there beyond the lower Rio Grande Valley. Interestingly, *Culex coronator*, *Culex declarator* and *Culex interrogator*, all now established non-native species in Florida, likely arrived in Florida via a recent north- and eastward expansion from southern Texas along the Gulf Coast [8,71,72], suggesting that environmental and climatic trends may facilitate geographic expansions across a Gulf Coast route. Additionally, major port cities along the Gulf Coast (Houston, New Orleans, Gulf Port, and Mobile) provide entry points that could further facilitate the geographic expansion of *Ae. scapularis*.

Until recently, *Ae. scapularis* was formerly known to occur across much of the Neotropics, including some Caribbean islands, but was not known from the Florida mainland. The recent collections of *Ae. scapularis* in Miami-Dade and Broward Counties [25], Florida suggest that *Ae. scapularis* could expand its mainland distribution northward and westward, and possibly into the southeastern United States, as no substantial geographic barriers limit its expansion except environmental unsuitability. While human-mediated transport is a likely culprit in the establishment of *Ae. scapularis* in mainland Florida, continued changes in abiotic environmental conditions and human-mediated habitats will be important to monitor, when considering further distributional expansions.

Our model predictions suggested that mosquito surveillance programs in Florida, particularly those along both coasts, should be aware of this species (Figure 6). Many Florida counties have robust mosquito control programs which actively trap mosquitoes in these areas [73]. Close inspection of *Aedes* Ochlerotatus Group specimens is warranted as *Ae. scapularis* could be misidentified as morphologically similar species known from Florida: *Aedes infirmatus* and *Aedes condolescens*. *Aedes condolescens* is limited to coastal areas of the southern peninsula, while *Ae. infirmatus* occurs statewide. The three species share a conspicuous patch of silvery or light-colored scales on the anterior scutum, but only *Ae. scapularis* has a stripe of pale scales on the hindtibia [74].

## 5. Conclusions

*Aedes scapularis* is a neotropical vector mosquito that transmits arboviruses and parasites of medical and veterinary importance. Its recent establishment in the southeastern peninsular Florida has potential public health implications, requiring active surveillance and utilization of modeling approaches to predict where the environment may be suitable for this species. The ecological niche model presented here provides a valuable tool to help inform vector control and public health surveillance efforts, characterizing areas that may be suitable for the geographic expansion of this species, while providing more detailed information about environmental suitability throughout its previously known distribution. As global connectivity continues to increase and environmental conditions continue to change, combining quantitative modeling approaches with field collected data will be critical to maximizing surveillance tools to monitor medically important mosquito vectors.

## Figures and Tables

**Table 1:** Bioclimatic variables included in candidate environmental data sets used to develop a model predicting the potential geographic distribution of *Ae. scapularis* in North and South America.

Variable Number	Variable Name
Bio1	Average Annual Temperature
Bio3	Average Isothermality (mean diurnal range/temperature annual range)
Bio5	Average Maximum Temperature of the Warmest Month
Bio6	Average Minimum Temperature of the Coldest Month
Bio12	Average Annual Specific Humidity
Bio16	Average Specific Humidity of the Wettest Quarter
Bio17	Average Specific Humidity of the Driest Quarter

**Table 2:** Variables included in the final model and percent contributions to model performance.

Variable	Variable Description	Percent Contribution
Bio3	Average Isothermality (mean diurnal range/temperature annual range)	48.6
Bio17	Average Specific Humidity of the Driest Quarter	22.3
Bio1	Average Annual Temperature	15.6
Bio5	Average Maximum Temperature of the Warmest Month	13.5

## Data Availability

Data is contained within the article or Supplementary Material.

## Supplementary Materials

The following are available online at https://www.mdpi.com/2075-4450/12/3/213/s1, Figure S1: Correlation plot of environmental variables, Figure S2: Map of raw occurrence data and thinned occurrence data, Table S1: Pearson’s correlation matrix of environmental variables, Table S2: Thinned occurrence data, Table S3: Full results of 595 candidate model sets, Table S4: Best candidate models, Figure S3: Histogram of Maholanobis distances and map of higher distance points, Figure S4: Area under the curve of the receiver operating characteristic (AUC) for best performing model, Figure S5: Plot of Maxent’s Multivariate Environmental Similarity Surface function to identify areas within the projection region with high potential for model extrapolation to combinations of environments not represented in the **M**-calibration region.
