# Supplementary material for: Potential Distribution of Aedes (Ochlerotatus) scapularis (Diptera: Culicidae): A Vector Mosquito New to the Florida Peninsula
Source: Insects. 2021 Mar 3;12(3):213. doi: 10.3390/insects12030213 (PMC8001964; doi:10.3390/insects12030213)
Supplement: Supplementary file 1 [file insects-12-00213-s001.zip › insects-1062954-supplementary/insects-1062954-supple-conversion/insects-1062954-supple-Figures.docx]

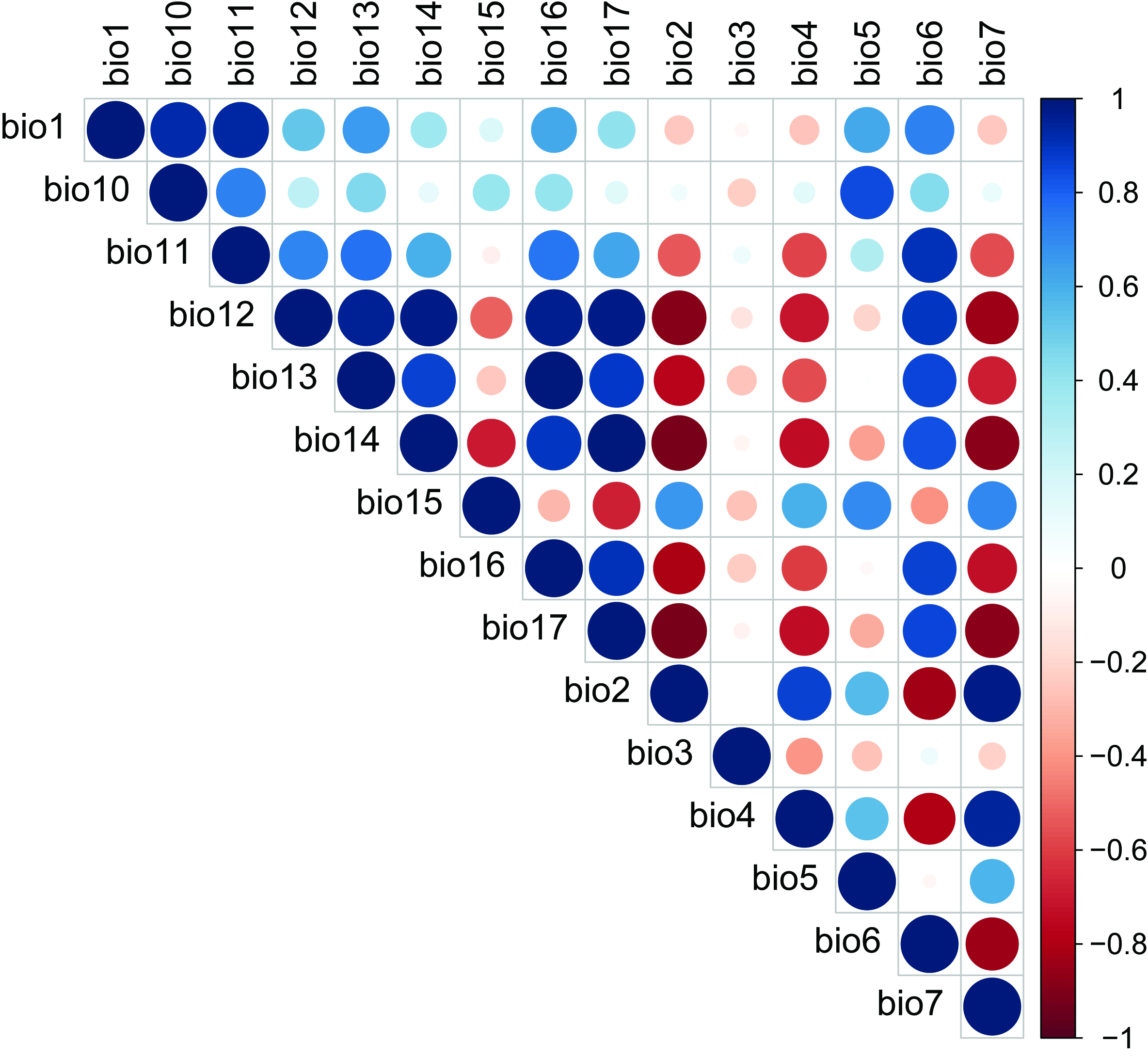


**Figure S1.** Correlation plot of environmental variables.

**Figure S2.** Map of raw occurrence data and thinned occurrence data.


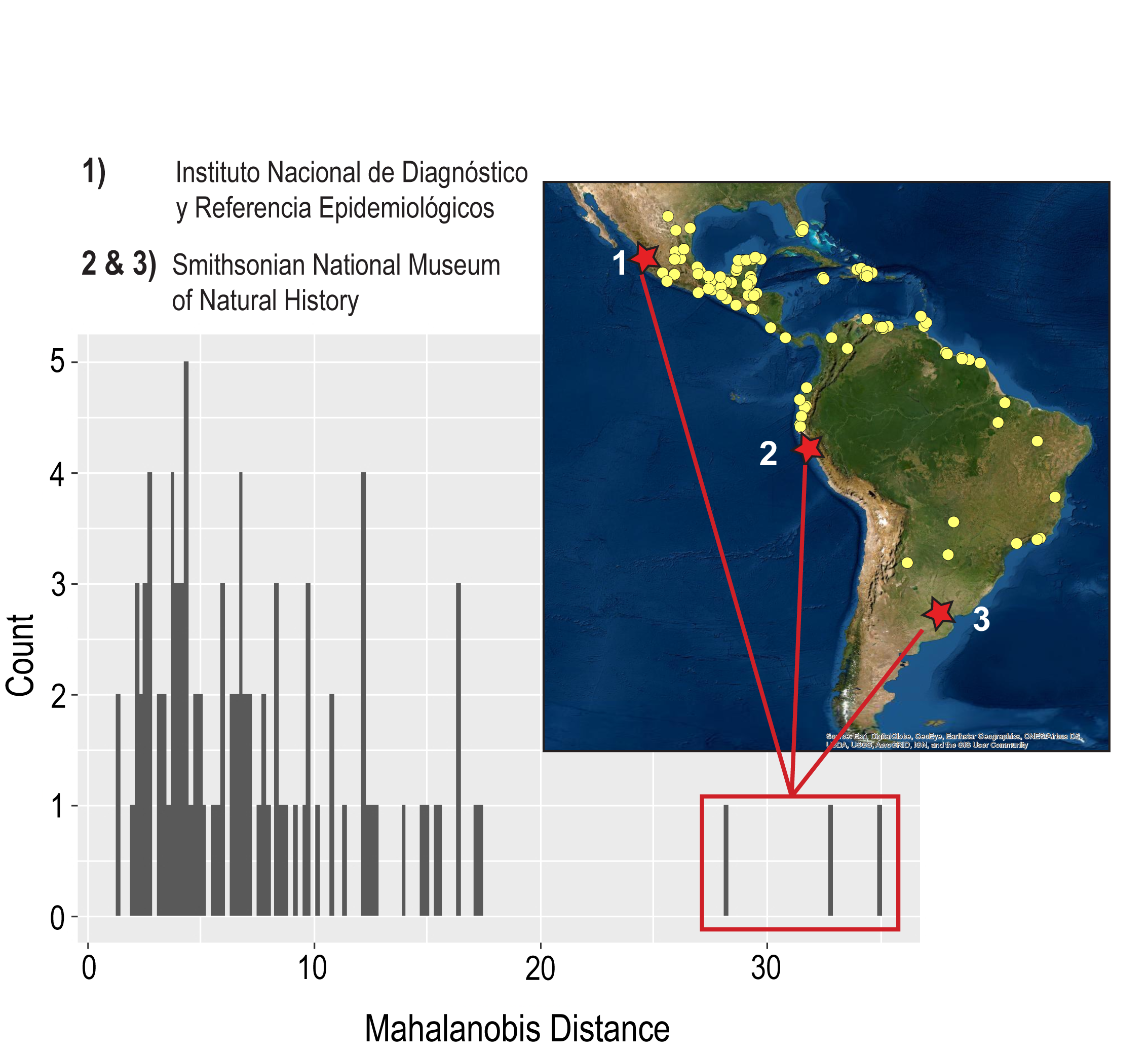


**Figure S3.** Histogram of Maholanobis distances and map of higher distance points.


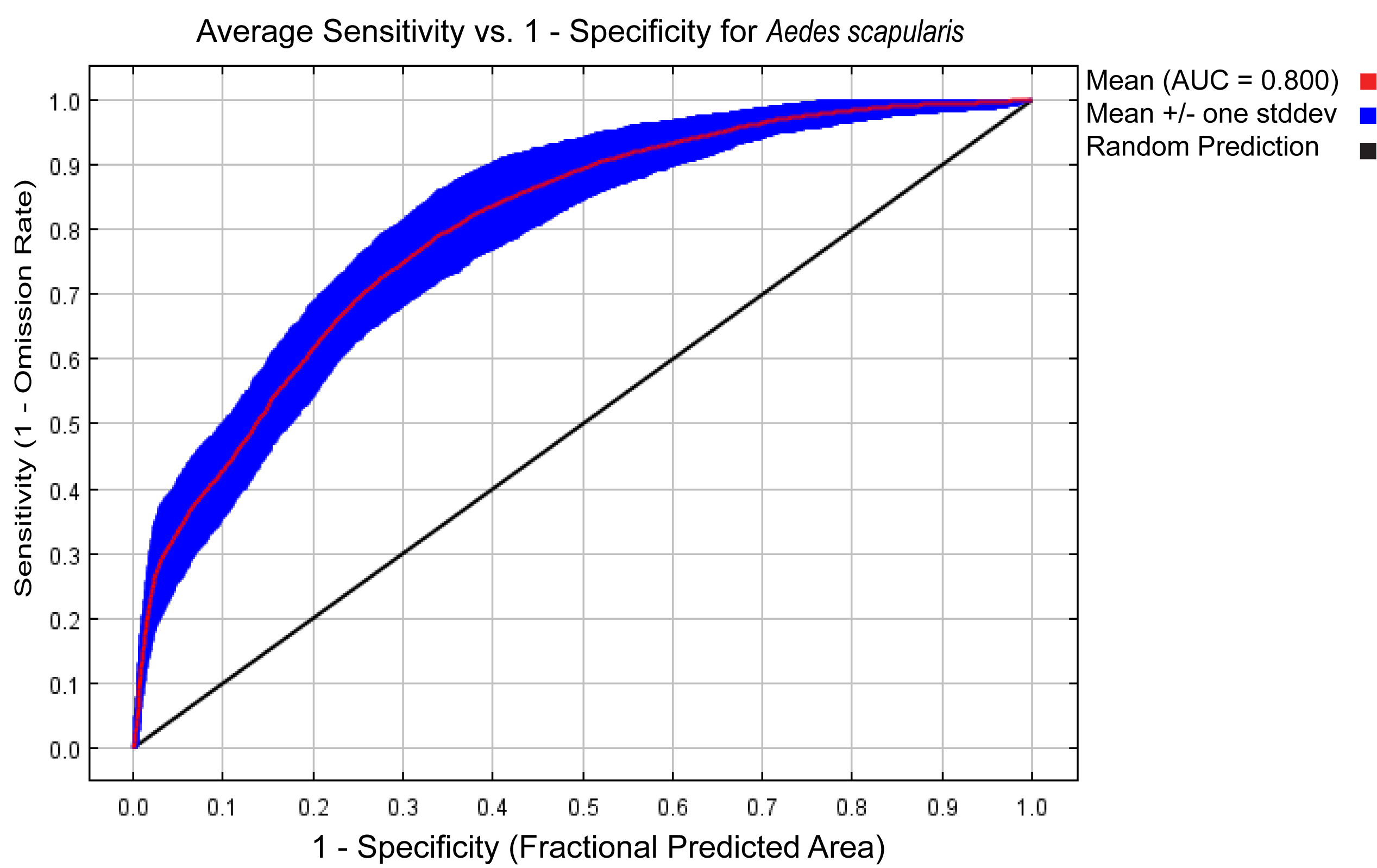


**Figure S4.** Area under the curve of the receiver operating characteristic (AUC) for best performing model.


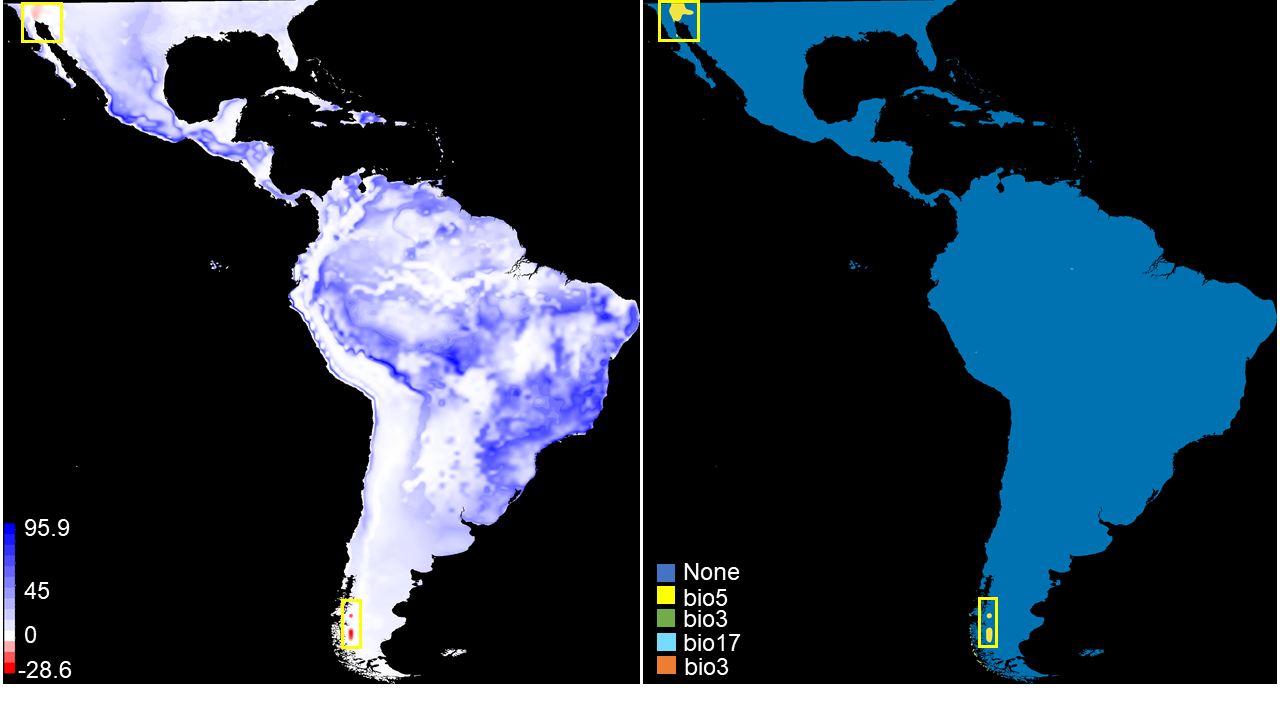


**Figure S5.** Plot of Maxent’s Multivariate Environmental Similarity Surface function to identify areas within the projection region with high potential for model extrapolation to combinations of environments not represented in the **M**-calibration region.
